# A Live-Cell Imaging-Based Fluorescent SARS-CoV-2 Neutralization Assay by Antibody-Mediated Blockage of Receptor Binding Domain-ACE2 Interaction

**DOI:** 10.3390/biotech14010010

**Published:** 2025-02-14

**Authors:** Jorge L. Arias-Arias, Laura Monturiol-Gross, Eugenia Corrales-Aguilar

**Affiliations:** 1 Centro de Investigación en Enfermedades Tropicales (CIET), Facultad de Microbiología, Universidad de Costa Rica, San José 11501-2060, Costa Rica; jorgeluis.arias@ucr.ac.cr; 2 Dulbecco Lab Studio, Residencial Lisboa 2G, Alajuela 20102, Costa Rica; 3 Instituto Clodomiro Picado (ICP), Facultad de Microbiología, Universidad de Costa Rica, San José 11501-2060, Costa Rica; laura.monturiol@ucr.ac.cr

**Keywords:** SARS-CoV-2, surrogate neutralization assay, fluorescence live-cell imaging, cell-based COVID-19 serology

## Abstract

Neutralization assays have become an important tool since the beginning of the COVID-19 pandemic for testing vaccine responses and therapeutic antibodies as well as for monitoring humoral immunity to SARS-CoV-2 in epidemiological studies. The spike glycoprotein (S) present on the viral surface contains a receptor binding domain (RBD) that recognizes the angiotensin-converting enzyme 2 receptor (ACE2) in host cells, allowing virus entry. The gold standard for determining SARS-CoV-2 neutralizing antibodies is the plaque reduction neutralization test (PRNT), which relies on live-virus replication performed exclusively in biosafety level 3 (BSL-3) laboratories. Here, we report the development of a surrogate live-cell imaging-based fluorescent SARS-CoV-2 neutralization assay, applicable to BSL-1 or BSL-2 laboratories, by antibody-mediated blockage of the interaction between recombinant RBD with overexpressed ACE2 receptor in a genetically modified HEK 293T stable cell line. Our approach was able to detect neutralizing antibodies both in COVID-19-positive human serum samples and polyclonal equine formulations against SARS-CoV-2. This new cell-based surrogate neutralization assay represents a virus-free fluorescence imaging alternative to the reported approaches, which can be used to detect antibody-neutralizing capabilities toward SARS-CoV-2. This assay could also be extrapolated in the future to other established and emergent viral agents.

## 1. Introduction

SARS-CoV-2, the causative agent of the coronavirus disease 2019 (COVID-19) [1], is an enveloped, single-stranded RNA virus, which mainly enters host cells through its obligate receptor, angiotensin-converting enzyme 2 (ACE2) [2,3,4,5,6,7,8,9]. Its mechanism for entry into cells has been recently reviewed [10]. The spike protein (S) encoded by the viral genome is the glycoprotein responsible for binding to the host receptor and mediates viral and host cellular membrane fusion, followed by the release of viral genomic RNA into the cell cytoplasm [10]. The S protein is assembled as a homotrimer and is inserted in multiple copies into the membrane of the virion [9,10]. The S protein contains a receptor binding domain (RBD) on its S1 subunit that specifically recognizes the ACE2 receptor [2,3,4,5,6,7]. Given its structural features, SARS-CoV-2 RBD bounds to ACE2 in a strong and dose-dependent manner [7,8].

Since SARS-CoV-2 RBD is the main specific viral structure that binds to the host cellular receptor, it has been an important tool for the development of vaccines, neutralizing antibodies, and assays for antibody detection [2,8,11,12]. In the last years, several approaches for recombinant production of RBD have been assessed [13,14,15,16,17,18,19,20]. Given its high specificity, this recombinant RBD has been widely used for the development and validation of serological assays, such as enzyme-linked immunosorbent assays (ELISA) [13,15,21,22,23,24] or surrogate neutralization assays [12,20,25], critical for monitoring immunological responses to SARS-CoV-2.

Among the multiple approaches for COVID-19 diagnosis, like PCR, CRISPR, aptamers, molecular imprinting, and microarray-based methods, antibody detection by serological assays plays a pivotal role as it also can be used for the monitoring of vaccine effectiveness and epidemiological surveillance [26,27,28]. Thus, detection of neutralizing antibodies has been performed since the first months of the SARS-CoV-2 pandemic. Relevant reviews about SARS-CoV-2 neutralization assays that encompass their broad use and development have been published [29,30,31]. The gold standard for determining neutralizing antibodies is the plaque reduction neutralization test (PRNT) [29,31,32,33,34,35]. Variations in this methodology, by using a reporter fluorescent virus (mNeonGreen SARS-CoV-2), have also been developed [36]. However, these approaches require a biosafety level 3 (BSL-3) laboratory, since they need live-virus replication. In consequence, several surrogate neutralizing antibody assays have been developed for their use in regular BSL-1 and BSL-2 laboratories. Within these, fluorescent pseudotyped viruses expressing SARS-CoV-2 S protein on a vesicular stomatitis virus (VSV) or human immunodeficiency virus type 1 (HIV-1) backbone have been established [21,29,31,37,38,39,40], as well as chemiluminescence immunoassay (CLIA) and ELISA-based neutralization assays that rely on ACE2-RBD interaction [12,20,25,29,31,41,42]. Although such approaches are great alternatives to PRNT, both are time-consuming and require either virological expertise or expensive reagents.

In the present study, we developed a fluorescence live-cell imaging-based surrogate neutralization assay that uses a recombinant SARS-CoV-2 RBD [15], and a genetically modified HEK 293T stable cell line co-expressing both the ACE2 receptor and the fluorescent protein dTomato, to detect neutralizing antibodies against SARS-CoV-2 in sera samples from COVID-19-positive patients and in polyclonal anti-SARS-CoV-2 equine formulations [43]. Our approach has the advantage of exploiting the metabolic machinery of the cell to directly synthesize, in a low safety level laboratory, both the RBD and the ACE2 receptor required for the assay. Moreover, the cellular co-expression of dTomato facilitates precise fluorescence normalization of the signal for a robust detection of RBD-ACE2 interactions. The same principle could be easily extrapolated to other viral infections by changing the genetic constructs used for the elaboration of the transgenic cell lines.

## 2. Materials and Methods

Molecular design and cloning. The coding sequence of the angiotensin-converting enzyme 2 (ACE2) from Homo sapiens (GenBank: NP_001358344.1) with the influenza virus hemagglutinin signal peptide (HA) at the N-terminus fragment followed by a c-Myc tag, was human codon-optimized, commercially synthesized and cloned (GenScript, Piscataway, NJ, USA) into the HindIII and XbaI sites of the transposon-based vector pSBbi-RP (a gift from Eric Kowarz, Addgene plasmid #60513) [44] to generate the plasmid pSBbi-RP-ACE2. The plasmid pSBbi-RP-CoV2/RBD-Puro (Addgene plasmid #161793) and its associated stable cell line HEK 293T RBD/dTomato for the recombinant production of the SARS-CoV-2 RBD (Wuhan-1) were previously developed [15]. Vector maps were generated with the software SnapGene Viewer 5.2.4 (Dotmatics, Boston, MA, USA).

Stable cell lines development. HEK 293T cells (ATCC, Manassas, VA, USA) were cultured in Dulbecco’s modified Eagle medium (DMEM, Gibco, Gaithersburg, MD, USA) supplemented with 10% FBS (Gibco), 1X GlutaMAX (Gibco), 1 mM sodium pyruvate (Gibco), and 1X antibiotic-antimycotic solution (Gibco), at 37 °C in an atmosphere of 5% CO_2_. The stable cell lines HEK 293T/dTomato and HEK 293T/dTomato/ACE2 were established by double transfection with Lipofectamine 3000 (Invitrogen, Carlsbad, CA, USA) of the vector pCMV(CAT)T7-SB100 codifying for the SB100X transposase (a gift from Zsuzsanna Izsvak, Addgene plasmid #34879) [45] and either the plasmid pSBbi-RP or the vector pSBbi-RP-ACE2, respectively. At 72 h post-transfection, cells were selected for 2 days with 2 μg/mL of puromycin (assay grade ≥ 98%, Sigma, St. Louis, MO, USA) in DMEM 10% FBS. The selected stable cell lines were grown and maintained in DMEM 10% FBS containing 0.25 μg/mL puromycin.

Direct immunofluorescence. The stable cell lines HEK 293T/dTomato and HEK 293T/dTomato/ACE2 were cultured on a µClear black 96-well plate (Greiner Bio-One, Monroe, NC, USA) at a density of 25,000 cells/well in DMEM 10% FBS and incubated overnight at 37 °C/5% CO_2_. Next, the medium was removed, and cells were fixed for 1 h with 50 µL/well of 1% paraformaldehyde (assay grade 95%, Sigma) at room temperature. After washing with 100 µL/well of PBS, direct immunofluorescence labeling of membrane c-Myc tagged ACE2 was performed with 50 µL/well of an anti-c-Myc-Alexa Fluor™ 647 tagged monoclonal IgG (Invitrogen, MA1-980-A647) during 1 h at 37 °C/5% CO_2_. Finally, cells were washed twice with 100 µL/well of PBS and imaged in 100 µL/well of FluoroBrite™ DMEM (Gibco) using a Lionheart FX automated fluorescence microscope (BioTek, Winooski, VT, USA) with the 20× objective and both the RFP (dTomato) and Cy5 (Alexa 647) filter/LED cubes.

Serum samples and ethics statement. Sera of SARS-CoV-2-positive patients were obtained after approval of the Ethical Board of the University of Costa Rica (CEC-164-2021). A cohort of 20 samples with positive serology by ELISA used in a previous study [15] were selected for the validation of the cellular neutralization assay. Sera of 20 anonymous pre-pandemic serum donors stored in the laboratory from a previous project (CEC-VI-3970-2013) were used as a SARS-CoV-2-negative pool. The anti-SARS-CoV-2 equine formulations (Anti-S1 and Anti-Mix) were provided by Instituto Clodomiro Picado, University of Costa Rica; an ethical statement of the development of equine formulations has already been included in a previous study [43].

Cellular Neutralization Assay. µClear black 96-well plates were incubated overnight at 4 °C with 40 µL/well of a 25 µg/mL fibronectin solution (Gibco, 33016-015) in PBS. Next, HEK 293T/dTomato/ACE2 cells stably expressing the fluorescent protein dTomato and the human ACE2 receptor were seeded at a density of 25,000 cells/well in DMEM 10% FBS and incubated overnight at 37 °C/5% CO_2_. Then, 2-fold serial dilutions of sera samples to be screened for neutralizing antibodies were incubated for 30 min at 37 °C with 0.12 µg/mL of recombinant SARS-CoV-2 RBD [15] in DMEM supplemented with 2% FBS. Sera previously screened by ELISA serology [15] from SARS-CoV-2-positive patients and the negative pre-pandemic pool were used as positive and negative controls, respectively. Next, 50 µL/well of the samples were added to the cells and incubated for 30 min at room temperature. After one wash with 100 µL/well of PBS, cells were incubated with 40 µL/well of 1/400 dilution of mouse monoclonal anti-6x-His tag Alexa Fluor™ 647-conjugated antibody (Invitrogen, MA1-135-A647) for 30 min at room temperature. Then, cells were washed twice with 100 µL/well of PBS and 100 µL/well of FluoroBrite™ DMEM supplemented with 2% FBS was added. Finally, cell images were acquired with a Lionheart FX automated fluorescence microscope using the 20× objective and both the RFP and Cy5 filter/LED cubes. Image analysis was performed with the software CellProfiler 4.0 (http://www.cellprofiler.org; Broad Institute, Cambridge, MA, USA, accessed on 22 March 2024). Interaction of the RBD with the ACE2 receptor generates a far-red dot-shaped fluorescence pattern when labeled with the anti-6x-His-Alexa 647 antibody. Therefore, the absence of this pattern when sera were screened denotes the presence of anti-RBD neutralizing antibodies in the samples.

## 3. Results

### 3.1. Stable Cell Lines Generation

**Figure 1:**
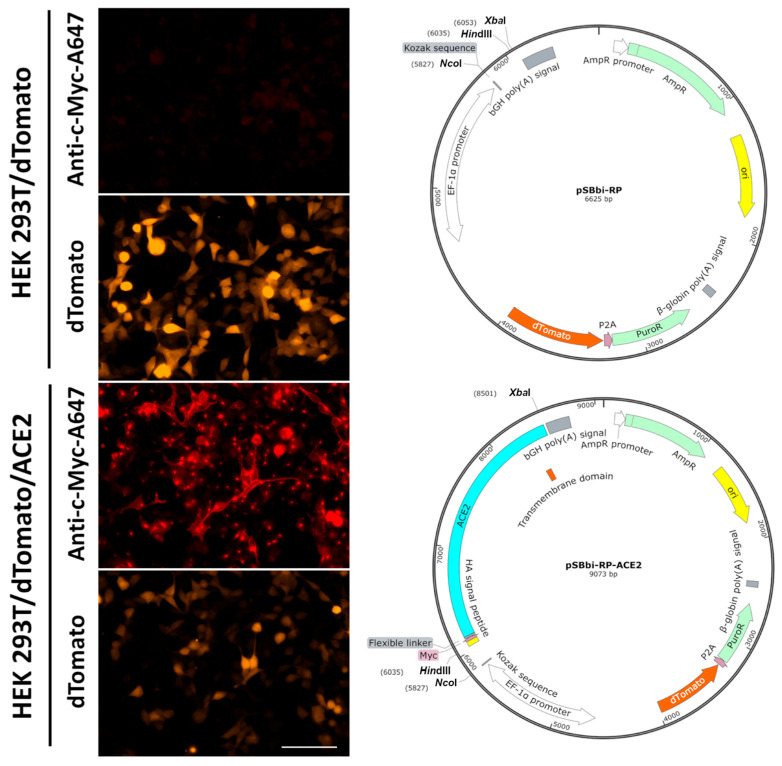
Stable cell lines used for the development of the SARS-CoV-2 cellular neutralization assay. HEK 293T/dTomato and HEK 293T/dTomato/ACE2 cells were generated by the transfection of the transposon vectors pSBbi-RP and pSBbi-RP-ACE2, respectively. These plasmids drive the expression of the fluorescent protein dTomato alone (orange) and both dTomato and a c-Myc-tagged version of the human ACE2 receptor, respectively. The confirmation of ACE2 expression on the cell membrane was determined by direct immunofluorescence on fixed non-permeabilized cells using an anti-c-Myc-Alexa 647 tagged antibody (red). Representative images from three independent experiments are shown. Total magnification of 200×, scale bar = 100 µm.

A stable HEK293/dTomato/ACE2 cell line was produced by transfecting wild-type cells with the transposon vector pSBbi-RP-ACE2 (Figure 1), which drives the co-expression of both fluorescent protein dTomato and c-Myc-tagged human ACE2 receptor. Simultaneously, a control cell line was generated by stable transfection of the vector pSBbi-RP (Figure 1), which drives the expression of dTomato alone. Accordingly, the expression of dTomato (orange) was observed in both stable cell lines, while membrane expression of ACE2 receptor, determined by an anti-c-Myc-Alexa 647 tagged antibody (red), was only seen in the stable HEK 293T/dTomato/ACE2 cells (Figure 1). After incubation with recombinant RBD, the functionality of ACE2 receptors was demonstrated by the overlapping of the fluorescence signals of conjugated antibodies used to label both the receptor and its attached ligand by direct immunofluorescence (Figure S1).

### 3.2. Determinations of Recombinant RBD Concentration for the Cellular Neutralization Assay

**Figure 2:**
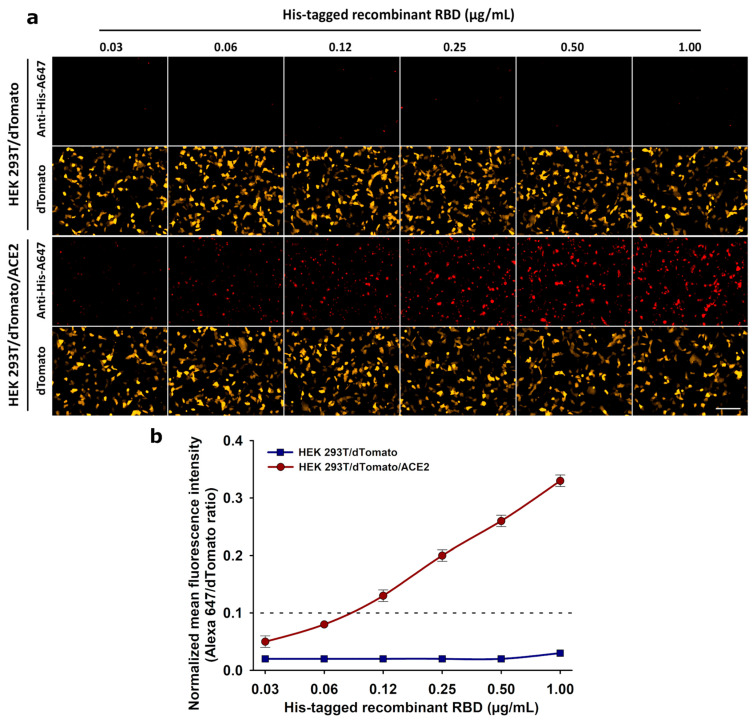
Determination of the optimal amount of recombinant RBD for the SARS-CoV-2 cellular neutralization assay: (**a**) Both HEK 293T/dTomato (negative control of ACE2 expression) and HEK 293T/dTomato/ACE2 cells were incubated with rising concentrations of His-tagged recombinant SARS-CoV-2 RBD in the range 0.03–1.00 µg/mL. Finally, the assay was revealed with an anti-His-Alexa 647-tagged antibody labeling (red). Representative images from three independent experiments are shown. Total magnification of 200×, scale bar = 100 µm. (**b**) Graphical data of the fluorescence intensity obtained by digital image analysis. A cut-off of 0.1 in the normalized fluorescence signal (Alexa 647/dTomato) was established for HEK 293T/dTomato/ACE2 cells as five times the mean value obtained with the same labeling in HEK 293T/dTomato control cells. Data are expressed as means ± S.D. of three independent experiments.

To optimize the amount of recombinant RBD for the neutralization assay, rising concentrations of His-tagged recombinant RBD generated as previously described [15] were incubated with both HEK 293T/dTomato (control) and HEK 293T/dTomato/ACE2 cells. By using an anti-His-Alexa 647-tagged antibody to reveal the RBD protein, a dot fluorescence pattern was observed, starting at 0.06 µg/mL concentration, due to its interaction with the ACE2 receptor present in HEK 293T/dTomato/ACE2 cells (Figure 2a). A cut-off value of 0.1 was established, as five times the mean value of the normalized fluorescence signal present in the HEK 293T/dTomato control cells (Figure 2b). Given the results, a concentration of 0.12 µg/mL of RBD recombinant protein was selected for the cellular neutralization assay. This represents the minimum concentration tested where the curve showed the best coefficient of determination (R^2^ = 0.96) of the fluorescence signal increase against the rising concentrations of RBD. In practical terms, 0.12 µg/mL represents a concentration that is low enough to be 100% neutralized by the patient’s antibodies (if present) and high enough to avoid faint reactions that could fall into a grey zone of interpretation. This could minimize both false-negative and false-positive outcomes, improving the sensitivity and specificity of the assay, respectively.

### 3.3. Evaluation of the Cellular Neutralization Assay with Human Serum Samples

**Figure 3:**
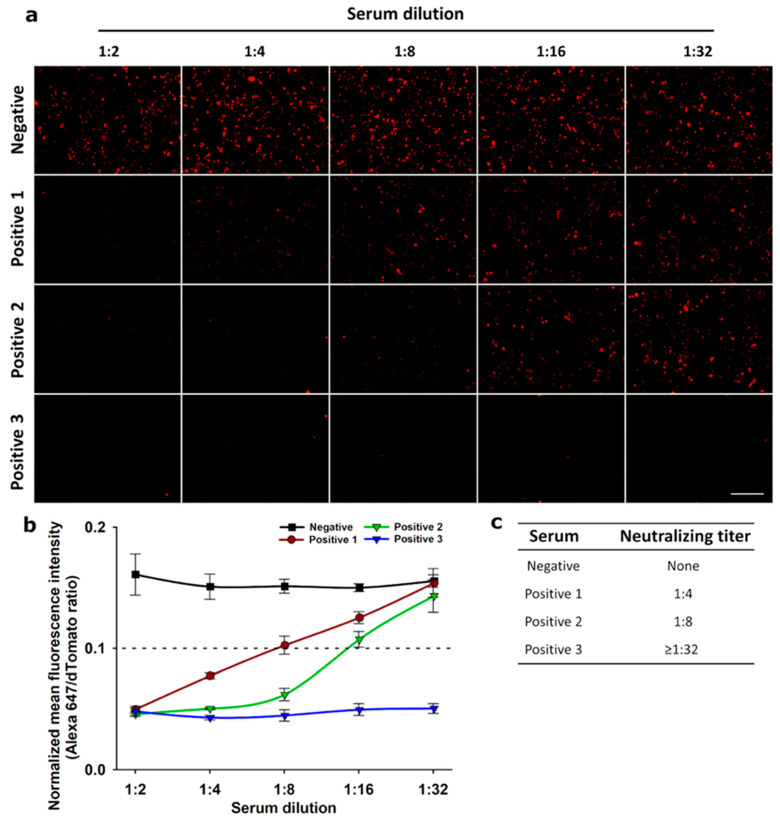
Evaluation of the SARS-CoV-2 cellular neutralization assay with human serum samples: (**a**) Cellular neutralization assay was applied to determine neutralizing antibodies titers in 2-fold serial dilutions of a negative pre-pandemic serum and three positive serum samples from SARS-CoV-2-positive patients defined by ELISA serology. Representative images from three independent experiments are shown. Total magnification of 200×, scale bar = 100 µm. (**b**) Data on the fluorescence intensity were obtained by digital image analysis and graphically visualized. Serum dilutions with a normalized fluorescence signal (Alexa 647/dTomato) below the previously defined cut-off of 0.1, were considered positive for the presence of neutralizing antibodies. Data are expressed as means ± S.D. of three independent experiments. (**c**) The neutralization titer for each tested serum was defined as the higher dilution with a normalized fluorescence signal below the cut-off.

We envisaged a new surrogate neutralization assay by incubating human serum samples from SARS-CoV-2-positive patients with recombinant His-tagged RBD to evaluate the presence of neutralizing antibodies by the blockage of RBD interaction with the recombinant ACE2 receptor present in stable cells (HEK 293T/dTomato/ACE2 cells). The pre-pandemic negative serum pool was not able to neutralize the recombinant His-tagged RBD binding to the cellular ACE2 receptor, generating a dot fluorescence pattern after labeling with an anti-His tag-Alexa 647-conjugated antibody (Figure 3a). On the contrary, serum from three SARS-CoV-2-positive patients, previously defined by ELISA serology, effectively blocked RBD binding to its receptor as evidenced by the absence of fluorescence (Figure 3a). Normalized fluorescence signal (A647/dTomato) below the previously defined cut-off of 0.1, was considered positive for the presence of neutralizing antibodies (Figure 3b). This surrogate neutralization assay also allows the calculation of a neutralizing titer for each tested serum, defined as the higher serum dilution that produces a normalized fluorescence signal below the cut-off (Figure 3c).

Comparison of the cellular neutralization assay against the results given by a previously developed ELISA in the same human serum samples denotes a direct positive correlation, as serum samples with higher absorbance values at 490nm in the colorimetric ELISA also resulted in higher neutralizing titers (Table 1).

### 3.4. Evaluation of the Cellular Neutralization Assay with Polyclonal Anti-SARS-CoV-2 Equine Formulations

**Figure 4:**
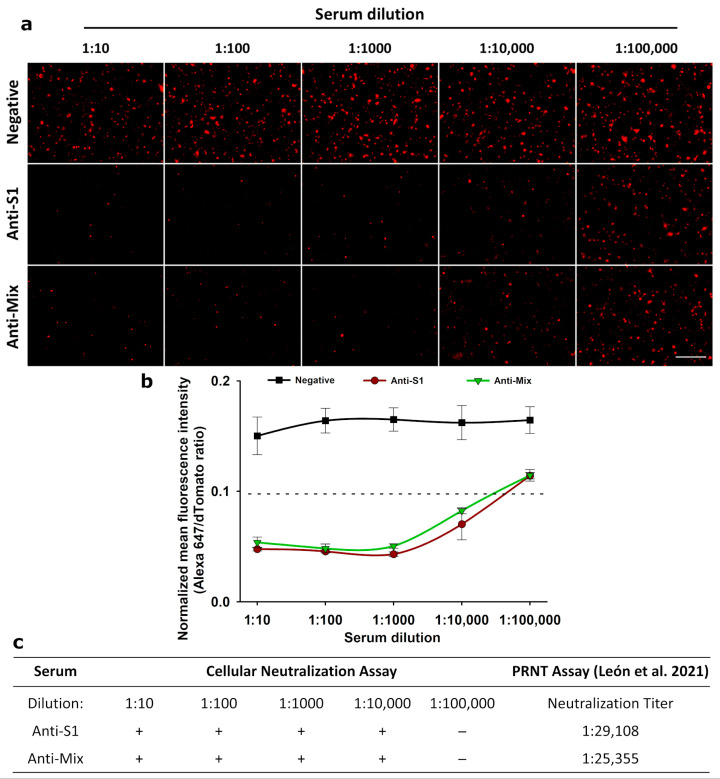
Evaluation of the SARS-CoV-2 cellular neutralization assay with two anti-SARS-CoV-2 polyclonal equine formulations: (**a**) The SARS-CoV-2 cellular neutralization assay was applied to determine the presence of neutralizing antibodies in 10-fold serial dilutions of a negative pre-pandemic serum pool and two anti-SARS-CoV-2 equine formulations. Representative images from three independent experiments are shown. Total magnification of 200×, scale bar = 100 µm. (**b**) Graphical data of the fluorescence intensity obtained by digital image analysis. Serum dilutions with a normalized fluorescence signal (Alexa 647/dTomato) below the previously defined cut-off of 0.1, were considered positive for the presence of neutralizing antibodies. Data are expressed as means ± S.D. of three independent experiments. (**c**) Consistent neutralization results were obtained when compared with data generated by PRNT performed on the same equine formulations in a previous study [43].

In addition, we tested the cellular neutralization assay with two previously characterized anti-SARS-CoV-2 equine formulations: an anti-S1 that consisted of a polyclonal antibody formulation developed from horses immunized with the recombinant S1 subunit from the S protein; and an anti-Mix, which is a polyclonal antibody formulation derived from horses immunized with a mixture of S1, N, and Spike-E-M mosaic recombinant proteins [43]. Both equine formulations were able to neutralize recombinant His-tagged RBD binding to the cellular ACE2 receptor at a 1:10,000 dilution, visualized by the absence of the dot fluorescence pattern present in the incubation with the pre-pandemic negative serum pool (Figure 4a). For both anti-S1 and anti-Mix formulations, the next 10-fold dilution (1:100,000) was considered negative for the presence of neutralizing antibodies, since normalized fluorescence signals fall above the previously defined cut-off of 0.1, establishing a neutralizing titer present at some point between the range 1:10,000–1:100,000 (Figure 4b). Data obtained with the infectious virus by PRNT using the same equine formulations showed neutralization titers of 1:29,108 and 1:25,355 for anti-S1 and anti-Mix formulations, respectively [43]. Hence, our surrogate neutralization assay, although determined by 10-fold serial dilutions, overlaps with a range that includes the titers obtained by the gold standard PRNT (Figure 4c).

## 4. Discussion

During the recent global SARS-CoV-2 pandemic, neutralization assays became an important tool for the development of interventional strategies against the virus such as convalescent plasma, vaccines, and monoclonal/polyclonal antibodies. Given their essential role in testing functional humoral immunity, they were also applied in surveillance epidemiological studies [12,20,38,46].

Detection of neutralizing antibodies against SARS-CoV-2 has been performed by different approaches in vitro. PRNT requires working with the infectious virus for several days in BSL-3 laboratory facilities. Therefore, other surrogate approaches such as pseudotyped virus expressing SARS-CoV-2 proteins [37,38,39] and immunoassay-based neutralization tests [12,20,41,42] have been developed for their application in BLS-1 or BLS-2 laboratories. However, both methodologies are time-consuming to complete and require either expertise in virological methods or costly reagents.

In the present study, we envisaged a fluorescent neutralization assay based on the SARS-CoV-2 RBD interaction with ACE2 receptor expressed on animal cells, instead of the colorimetric ELISA and chemiluminescent CLIA reported by others [12,20,42]. For this assay, a new stable cell line was developed that simultaneously co-expresses the fluorescent protein dTomato and a c-Myc-tagged version of the human ACE2 receptor, giving the opportunity to detect transfected cells easily by d-Tomato orange fluorescence and at the same time allowing the expression of ACE2 receptor with an anti-c-Myc antibody to be corroborated (Figure 1). This approach uses a recombinant SARS-CoV-2 His-tagged RBD (Wuhan-1) [15], whose quantity was optimized for the neutralization assay (Figure 2). When pre-incubating this SARS-CoV-2 RBD with serum from patients, neutralizing antibodies (if present) bind to it, blocking its interaction with the ACE2 receptor expressed on the stable cell line HEK 293T/dTomato/ACE2 (Figure 3 and Figure 4). Results of the assay are revealed with a fluorescent anti-His tag antibody that recognizes the recombinant His-tagged RBD protein when attached to the cellular ACE2 receptor, generating a dot fluorescence pattern. Therefore, the absence of this pattern when screening both human (Figure 3) or equine sera (Figure 4) denoted the presence of neutralizing antibodies in the samples. Although this assay was assessed for Wuhan-1 RBD, the coding sequence of RBD can be further changed to generate different versions for other clinically relevant SARS-CoV-2 variants.

Among the advantages of our new approach, the co-expression of the ACE2 receptor and the fluorescent protein dTomato by the same bicistronic promoter allows the fluorescence given by the anti-His-Alexa 647 fluorescent readout (Alexa 647/dTomato radio) to be directly normalized in the cells for a more robust detection of the RBD-ACE2 interactions. This is very useful due to the differential levels of ACE2 expression among the population of cells of the stable cell line. Moreover, as the quantification of fluorescence is performed by image analysis, our assay has the advantage of giving more objective results when compared to manually observed pseudotyped virus fluorescence, which is limited by the skills of the analyst. Another advantage is that the fluorescent readout has higher sensitivity when compared to colorimetric surrogate ELISAs [47,48].

Previous studies with SARS-CoV-1 and MERS have shown that neutralizing antibodies are not only directed to RBD but also toward the S1-N-terminal domain (S1-NTD) and the S2 region, blocking not only the binding of RBDs to their respective receptors but also interfering with S2-mediated membrane fusion and entry into the host cell [49,50]. Therefore, our assay presents the drawback of not considering other neutralizing targets in the S protein that could also be involved in viral neutralization, as discussed by others who also developed neutralization assays that rely on RBD-ACE2 interaction [20].

Moreover, when comparing the ELISA absorbance results of the same human sera with titers obtained by our new cellular neutralization assay, a good correlation was observed (Table 1). Other studies that compared both kinds of methods have also found a correlation between IgG antibodies measured by ELISA and neutralizing antibody titers [34,38]. A systematic review and meta-analysis study that investigated neutralizing antibodies in correlation with IgG of individuals with former SARS-CoV-2 infections found a better correlation for the data of each individual study that conformed to the subset analyzed [46]. However, the correlation between the detection of IgG and neutralizing antibodies at the whole meta-analysis level was low. This can be explained by a high variation between individual studies, attributed to the different neutralization and ELISA assays used [46].

Here, we demonstrate that the cellular neutralization approach was useful in detecting neutralizing antibodies in both human serum samples (Figure 3) and anti-SARS-CoV-2 equine formulations (Figure 4). When compared with the gold standard PRNT in the same equine formulations, we obtained neutralization titers in the same range as our surrogate test, pointing to its robust detection capabilities of neutralizing antibodies both in human and animal samples. Others have also achieved a good correlation between their surrogated neutralization assay and the PRNT approach [20,34].

Furthermore, our new surrogate method has the great biotechnological advantage of utilizing the metabolism of animal cells for the synthesis of key components of the assay: recombinant RBD and ACE2 receptor, under low safety levels laboratory conditions. Since SARS-CoV-2 is still circulating among humans/animals, and remains a public health concern, this assay can be further validated for clinical or research applications and be useful for monitoring protective neutralizing titers in patients, putative hosts, vaccinated individuals in high-risk populations, and for the efficacy evaluation of new vaccine candidates and therapeutics based on the development of antibodies against SARS-CoV-2.

Finally, this assay was developed considering the straightforward adaptability of the system to other diseases by easily modifying the coding vectors used for the elaboration of the stable cell lines that produce the antigens, as the readout antibody is directed toward the generic His-tag included in the same recombinant proteins. Thus, our approach could be relevant to monitor humoral neutralizing responses in future scenarios due to the emergence of new etiological agents that humanity is facing due to the ongoing sociopolitical and climatic crisis.

## Figures and Tables

**Table 1:** Comparison of the SARS-CoV-2 cellular neutralization assay and ELISA * for COVID-19 serology.

Serum ID	ELISA Serology Assay (Mean OD490 nm)	Cellular Neutralization Assay (Neutralizing Titer)
cov20	0.47 ± 0.04	1:8
cov24	0.73 ± 0.02	≥1:32
cov41	0.66 ± 0.02	≥1:32
cov60	0.64 ± 0.03	1:16
cov142	0.61 ± 0.03	1:16
cov163	0.78 ± 0.02	≥1:32
cov170	0.41 ± 0.02	1:4
cov11-2	0.77 ± 0.03	≥1:32
cov168-2	0.51 ± 0.02	1:8
cov250	0.47 ± 0.02	1:4
cov262	0.40 ± 0.01	1:2
cov288	0.65 ± 0.01	≥1:32
cov290	0.57 ± 0.07	1:16
cov291	0.53 ± 0.01	1:8
cov292	0.44 ± 0.01	1:4
cov293	0.58 ± 0.01	1:8
cov298	0.34 ± 0.01	1:2
cov301	0.77 ± 0.01	≥1:32
cov45-3	0.31 ± 0.01	1:2
cov317	0.75 ± 0.01	≥1:32
Negative	0.21 ± 0.01	None

* [15].

## Data Availability

The original contributions presented in this study are included in the article/supplementary material. Further inquiries can be directed to the corresponding author(s).

## Supplementary Materials

The following supporting information can be downloaded at: https://www.mdpi.com/article/10.3390/biotech14010010/s1, Figure S1: Functional validation of recombinant ACE2 receptors stably expressed on HEK 293T cells.
