# Supplementary material for: A Live-Cell Imaging-Based Fluorescent SARS-CoV-2 Neutralization Assay by Antibody-Mediated Blockage of Receptor Binding Domain-ACE2 Interaction
Source: BioTech (Basel). 2025 Feb 14;14(1):10. doi: 10.3390/biotech14010010 (PMC11843899; doi:10.3390/biotech14010010)
Supplement: Supplementary file 1 [file biotech-14-00010-s001.zip › biotech-3385821-supplementary.pdf]

A Live-Cell Imaging-Based Fluorescent SARS-CoV-2 Neutralization Assay by Antibody-Mediated Blockage of Receptor Binding Domain-ACE2 Interaction

Jorge L. Arias-Arias [1,2], Laura Monturiol-Gross [3] and Eugenia Corrales-Aguilar [1,*]

[1] Centro de Investigación en Enfermedades Tropicales (CIET), Facultad de Microbiología, Universidad de Costa Rica, San José 11501-2060, Costa Rica; jorgeluis.arias@ucr.ac.cr
[2] Dulbecco Lab Studio, Residencial Lisboa 2G, Alajuela 20102, Costa Rica
[3] Instituto Clodomiro Picado (ICP), Facultad de Microbiología, Universidad de Costa Rica,
San José 11501-2060, Costa Rica; laura.monturiol@ucr.ac.cr
* Correspondence: eugenia.corrales@ucr.ac.cr

Supplementary Figure S1

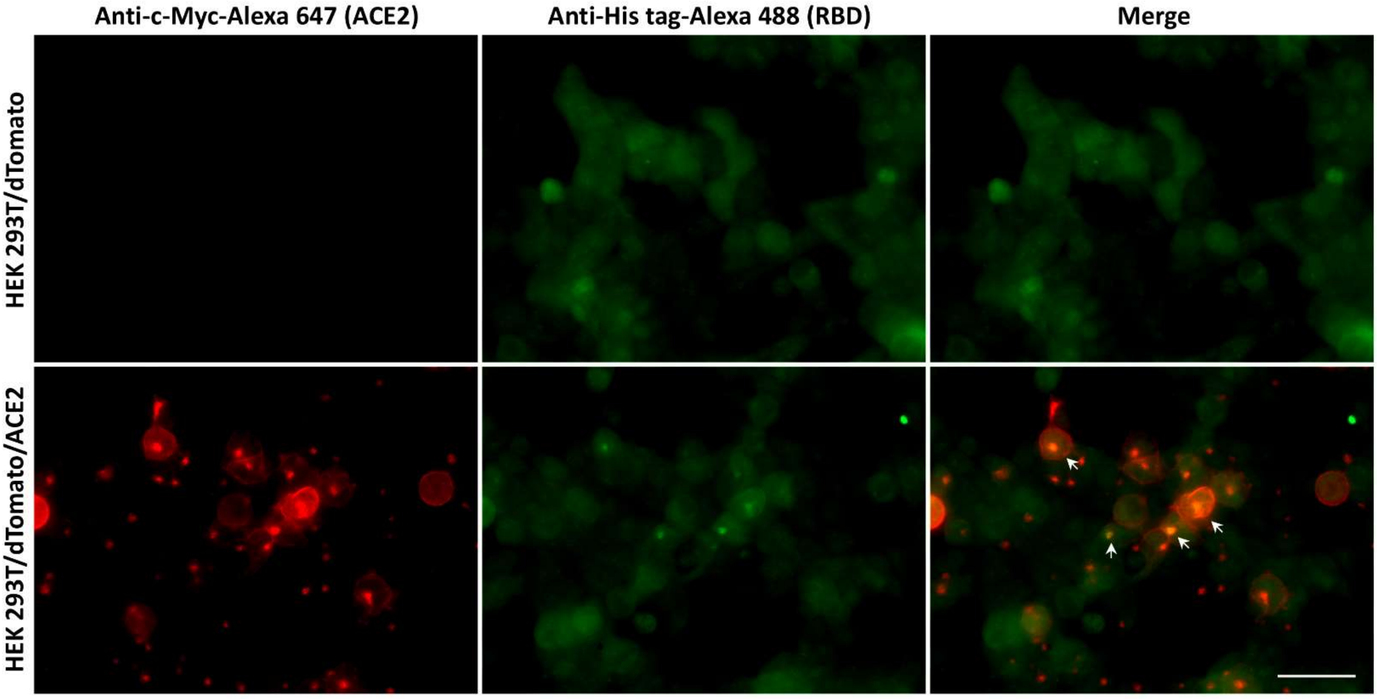

Figure S1. Functional validation of recombinant ACE2 receptors stably expressed on HEK 293T cells. To test the capacity of ACE2 receptors to interact with SARS-CoV-2 RBD, both HEK 293T/dTomato and HEK 293T/dTomato/ACE2 cells were incubated with conditioned medium containing his-tagged RBD from stable HEK 293T RBD/dTomato cells. After incubation, cells were fixed with 3.5% paraformaldehyde and both the c-Myc-tagged ACE2 receptor and the his-tagged RBD were labeled by direct immunofluorescence with anti-c-Myc-Alexa Fluor™ 647 (red) and anti-6x-His tag Alexa Fluor™ 488 (green, Invitrogen, MA1-21315-A488)-conjugated antibodies, respectively. Arrows show zones where both fluorescent signals overlap. Total magnification of 400X, scale bar = 50 µm.
